# Cytokine-induced killer cells: A novel immunotherapy strategy for leukemia

**DOI:** 10.3892/ol.2014.2780

**Published:** 2014-12-08

**Authors:** XIN-YU YANG, HUI ZENG, FANG-PING CHEN

**Affiliations:** 1 Depatment of Haematology, The Third Xiangya Hospital, Central South University, Changsha, Hunan 410013, P.R. China; 2 Department of Hematology, Xiangya Hospital, Central South University, Changsha, Hunan 410008, P.R. China

**Keywords:** cytokine-induced killer cells, adoptive immunotherapy, leukemia

## Abstract

Cytokine-induced killer (CIK) cells are NK-like T cells derived from peripheral blood mononuclear cells that are co-stimulated and expanded using cytokines for 14–21 days *in vitro*. CIK cells are a heterogeneous subset of highly-efficient cytotoxic T effector cells that mediate major histocompatibility complex-unrestricted cytotoxicity against a broad array of tumor cells. These effector cells are generated from patients with leukemia or healthy donors who demonstrate similar cytotoxic activity against leukemia blasts. Allogeneic CIK cells retain the ability to produce the graft versus tumor response and generate minimal graft versus host disease. In addition, CIK cells possess no cytotoxicity against normal hematopoietic stem cells *in vivo*. Leukemia recurrence remains a formidable obstacle, but adoptive immunotherapy offers promise for the eradication of minimal residual disease and prevention of leukemia relapse following hematopoietic stem cell transplantation. CIK cell infusion started a novel generation of adoptive immunotherapy and exhibits particular potential applications in the area of hematological malignancy. In the present study, the previous strategies of leukemia immunotherapy using CIK cells are reviewed and the future directions of development are discussed.

## 1. Research background and characteristics of CIK cells

Over the previous 20 years, the treatment of leukemia by chemotherapy or targeted therapies has achieved notable successes and even resulted in patients with long-term disease-free survival (1). However, the issue of minimal residual disease (MRD) and drug resistance in leukemia has plagued hematologists. Hematopoietic stem cell transplantation (HSCT), which was initially considered the best strategy for the radical treatment of leukemia and other hematological malignancies, was an exciting development and opened a novel era for the treatment of leukemia. However, for all the aforementioned treatment methods, a high relapse rate due to MRD remains the leading cause of treatment failure and the main cause of mortality. Currently, it appears unlikely that a single modality therapy may be effective for high-risk or advanced leukemia (2).

Novel and specific treatment strategies and techniques, including consolidation and salvage chemotherapy, HSCT and immunotherapy, have been eagerly used to prevent leukemia relapse when the tumor burden is at low levels (3,4). Animal models of acute myeloid leukemia (AML) have been demonstrated to be beneficial as they provided the basis for adoptive T and natural killer (NK) cell therapies (5). Passive immunotherapy strategies and, in particular, a novel form of adoptive immunotherapy have gained additional attention as the evidence from a clinical trial has demonstrated that cellular immunotherapy for the treatment of refractory malignancies played an important role. These cellular immunotherapies included donor lymphocyte infusion (DLI), which was demonstrated to be an effective strategy for adoptive cellular immunotherapy and exhibited evident anti-leukemia reactivity (6,7). Clinically, a sufficient number of effector cells that were safe and possessed robust cytolytic activity against leukemia blasts were sought, alongside the investigation into solving the problem of generating sufficient numbers of cytotoxic T lymphocytes (CTLs) or NK cells with powerful anti-leukemia activity, whilst continuing to exhibit a low incidence of graft versus host disease (GVHD), as required allogeneic CTL effector cells were also required (8).

In 1991, Schmidt-Wolf *et al* initially identified a heterogeneous cell population, the majority of which coexpressed cluster of differentiation (CD)3 and CD56 antigens, that activated and expanded from peripheral blood mononuclear cells in the presence of interferon (IFN)-γ, anti-CD3 monoclonal antibody and interleukin-2 (IL-2) *in vitro*. Ultimately, these cells developed to polyclonal T lymphocytes that shared a NK phenotype and function (9). This heterogeneous population was termed cytokine-induced killer (CIK) cells. CIK cells are extremely similar to natural killer T (NKT) cells, which are a subpopulation of T cells that also coexpress the CD3 and CD56 antigens. NKT cells play a vital role in the field of immunoregulation and anti-tumor immunity (10), and human type I NKT cells express a T cell receptor (TCR) composed of an invariant Vα24-JαQ chain in combination with several Vβ chains. By comparison with other T cells, a previous study identified that the CIK cells may be type II NKT cells due to the expression of a TCR without routine expression of the Vα24 ligand (11). CIK cells were found to mediate cytotoxicity against a broad array of tumor cells, and the killing mechanism was major histocompatibility complex (MHC)-unrestricted, with the CD3^+^/CD56^+^ cells being considered the main effector cells and exhibiting a greater antitumor activity compared with other cells (12,13). The MHC-unrestricted killing potential of the CIK cells aids in the reduction of tumor immune evasion and increase the killing efficiency.

Studies have identified that the effect cells of CIK cells terminal differentiation of CD3^+^/CD56^+^ fraction, while the CD3^+^/CD56^−^ CIK cells that also coexpressed the CD27, CD28 and CD62L antigens established earlier memory characteristics and reserved greater proliferative potential. Therefore, the amplification of effector cells is mainly derived from the initial stage of CD3^+^/CD56^−^ cells (14). Franchetti *et al* subsequently demonstrated that CIK cells, which were expanded from the initial phase of CD3^+^/CD56^−^/CD8^+^ T cells, expressed polyclonal T-cell receptor Vβ chains, the CD56 antigen, natural killer group 2, member D (NKG2D) and large granular lymphocyte morphology during terminal differentiation. However, the cells lacked expression of the majority of the NK-specific activating receptors, including NK cell p30-related protein (NKp30), NK cell p44-related protein and NK cell p46-related protein, and inhibitory receptors, such as killer-cell immunoglobulin-like receptor (KIR)2DL1, KIR2DL2, KIR3DL1, NKG2, member A and CD94 (15). At present, the exact cytotoxicity mechanism of CIK cells is not completely understood, but the NKG2D molecule expressed on the membrane cells is known to play a key role as the NKG2D molecule interacts with MHC-unrestricted ligands on tumor cells and the final kill role of the cell components was perforin and granzyme (16). Linn *et al* used oligonucleotide arrays to study the underlying molecular mechanism when CIK cells exerted anti-AML and anti-acute lymphocytic leukemia (anti-ALL) cell effects. It was found that the NK cell receptor genes NKG2, member C and NKG2, member E, together with perforin, were markedly upregulated. By contrast, a cytokine with immune inhibitory function, transforming growth factor β1, was markedly upregulated in the CIK cells that were cytotoxic to ALL cells, which may be the cause of the resistance of ALL cells to CIK cells (17).

Notably, previous studies have revealed that the CIK cells maintain dual-functional capability of NK and T cells, which transfer signals not only through TCR/CD3, but also through NKG2D, DNAX accessory molecule-1 and NKp30. These signal transmissions lead to the activation and secretion of CIK cells. In particular, the lymphocyte function-associated antigen-1 and Fas/FasL ligand are crucial in this process and also in the possible mechanisms involving in oncolytic activity of CIK cells (18,19). The dual-functional capability of CD3^+^/CD56^+^ CIK cells was found to be significantly impaired when administered with antibodies that respectively blocked these antigens or receptors.

The function of CIK cells generated from patients or donors exhibit the same cell activity *in vitro* and associated studies have reported that the cells exhibit an extremely high anti-tumor activity in several leukemia systems, including AML, ALL (20,21), chronic lymphocytic leukemia (22) and chronic myeloid leukemia (CML) (23). Furthermore, it is important to be aware that CIK cells demonstrate no cytotoxicity against normal CD34^+^ hematopoietic progenitor cells, with this characteristic of the cells allowing for a possible use in clinical immunotherapy (22). Nevertheless, the cytotoxicity of CIK cells against ALL cells was much lower compared with the other types of leukemia blasts (22). Subsequently, it was revealed that allogeneic CIK cells maintained vigorous graft-versus-tumor (GVT) activity and reduced MRD in the follow-up *in vivo* experiment (22). Nishimura *et al* evaluated the transportation and survival of luciferase-expressing CIK cells using a bioluminescent imaging technique in an allogeneic bone marrow transplant model. In this study, it was found that CIK cells produced a low risk of GVHD, likely due to the target organs lacking expression of the NKG2D ligands that were recognized by effector cells. The CIK cells gathered around the tumor cells until the tumor cells were eradicated following infusion (24). The mechanism behind the infusion of allogeneic CIK cells not developing GVHD was not a fully-understood phenomenon. Certain studies have identified that the deletion of CD3^+^/CD56^−^ CIK cells may result in no GVHD at all and speculated that CIK cells had the potential to separate GVT effects from GVHD (21). Fujiki *et al* also demonstrated that allogeneic CIK cells exhibited a low risk of developing GVHD due to the elimination of the dendritic cells (DCs) in the host by IFN-γ-secreting effector cells (26). These aforementioned findings provided a novel therapy method for patients with leukemia who relapsed following allogeneic HSCT (9,28–31). In comparison to lymphokine-activated killer (LAK) cells, CTLs, CD3-AK cells, NK cells and tumor-infiltrating lymphocytes, the CIK cells were the most promising effector cells for immunotherapy as the other cells possessed lower anti-tumor cytotoxic activity, were challenging to obtain in sufficient numbers or potentially caused fatal GVHD (Table I) (9,28–31). The characteristics of CIK cells are in high demand in the area of leukemia immunotherapy, which is one of the challenges that are now being faced in clinical treatment.

## 2. Characteristics of immunotherapy for leukemia using CIK cells

Passive immunotherapy includes immunomodulatory monoclonal antibodies, immune cells and cytokines. The concept of ‘immunoediting’ is associated with the manner in which tumors manipulate their microenvironment through tumor-derived cytokines, chemokines and other soluble factors. Therefore, once tumors have become clinically detectable, they have already evolved mechanisms to evade the immune response mounted by the host, which must be overcome to create effective and durable anti-tumor immunity. Therefore, the aim of adoptive immune cell therapy is how to obtain sufficient expansion of the special localization of effector cells. These methods of passive immunotherapy and active immunotherapy vary, but the immune mechanism demonstrates similar features between the two, also exhibiting a complex connection. It is known that the combination of these strategies is an improved hypothesis for increasing the efficacy of immunotherapy compared with the use of only one, if the combined strategies do not cause various severe autoimmune diseases (32). The current tactic for immunotherapy in leukemia is to combine these methods with monoclonal antibodies, vaccines or immune cell therapy to reduce the rate of relapse.

At present, it is challenging to accurately evaluate the final clinical appearance in leukemia immunotherapy using CIK cells. However, there is currently no evidence for the efficacy of CIK cells in the treatment of ALL. Therefore, it is necessary for novel approaches for the manipulation of CIK cells or target cells to be explored to further increase the anti-leukemic potency of CIK cells. In previous years, paradoxical effects of cytokines in tumor immune surveillance and tumor immune escape have been identified. Initially, studies aimed to improve the cytotoxicity of CIK cells by adding various cytokines that regulated of immune responses. Subsequently, manipulation of the CIK cells that expressed various chimeric artificial antigens or receptors was also attempted. Additionally, as aforementioned, the CIK cells were also targeted to tumor cells through NKG2D, which was an important identification signal in anti-tumor mechanisms of NK cells and CTLs. Therefore, through enhancing the expression of the NKG2D ligand, the effect of immunotherapy for cancer can be improved (31). MHC class I chain-related genes (MIC)A and MICB are important regions of the NKG2D ligands that are expressed in human cancer cells, Huang *et al* demonstrated that a combination of histone deacetylase inhibitors, to upregulate cell surface MICA/B in certain tumors, with metalloproteinase inhibitors to block MICA/B shedding can regulate cell-surface MICA/B levels, and eventually lead to significantly enhanced the anti-tumor activity of CIK cells (33). Dendritic cells (DCs) are the most effective antigen-presenting cells. DC-CIK cells can further enhance the antitumor activity against leukemia blasts. Furthermore, compared with CIK cells and DC-CIK cells, semi-allogeneic DC-CIK cells increased the proportion of CD3^+^/CD56^+^ cells and significantly augmented the antitumor activity (34,35). Table II (36–48) reports a series of studies that aimed to increase the cytotoxicity of CIK cells against leukemia cells using various strategies. In addition to those presented in Table II, the studies used other strategies to define and design CIK cells, but the studies were not performed using leukemia blasts. For instance, Yang *et al* increased the anti-tumor potency through the oncolytic adenovirus-mediated transfer of the human interleukin-12 (hIL-12) gene into CIK cells, which secreted a high level of hIL-12 that improved the anti-tumor potency of the cells (49).

The potential of CIK cells in combination with vaccination and chimeric antigen receptors (CARs) in cancer immunotherapy in significant. Immune cells have been demonstrated to substantially improve the response tumor vaccines to immunotherapy. CIK cells may also possess potential therapeutic benefits in the process of long-term tumor control. Leukemia stem cells (LSCs) initiate and sustain the clonal hierarchy of leukemia blasts and possess biological properties rendering the LSCs resistant to conventional chemotherapy. The CD19, CD33 and CD123 antigens are strongly expressed in various leukemic blasts and LSCs. LSCs may be identified and targeted by various surface markers and therefore, a lentiviral vector expressing a CARs was designed, including anti-CD19 chimeric receptor, anti-CD33 chimeric receptor and anti-CD123 chimeric receptor, and transfected into CIK cells, which then specifically targeted primary leukemia cells or LSCs. Previously, the potency of the CAR approach in redirecting CIK cells against acute leukemia cells has been revealed and the present study highlighted, as demonstrated by previous studies, the crucial role exerted by co-stimulatory molecules in the CAR signaling domain, which significantly improved the anti-tumor effector function of CAR-expressing CIK cells (41–43).

## 3. Clinical trials of CIK cell infusion for leukemia therapy

During the past decade, attempts were made to adopt CIK cell infusion for immunotherapy to treat leukemia when it was identified that CIK cell infusion exhibited greater antitumor activity compared with LAK cells. Niam *et al* initially reported that the clinical application of CIK cells derived from health donors and patients with AML and CML, at various stages of therapy, was feasible and safe (50). This was encouraging and accelerated the application of CIK cells for clinical trial. Introna *et al* subsequently reported a phase I study of CIK cell immunotherapy that repeated the infusion of allogeneic CIK cells into patients. The subjects included four patients with AML, three patients with host disease (HD), one patient with CML, one patient with pre-B ALL and two patients with myelodysplasia (MDS), who all relapsed following allo-HSCT. Prior to the infusion of CIK cells, six patients had received other salvage treatments, including chemotherapy, radiotherapy and DLI. However, the patients had not exhibited any significant response to therapy. The median number of CIK infusions and total CIK cells was two (range, 1–7) and 12.4×10^6^/kg (range, 7.2–87.4), respectively. The infusions were administered until the patients achieved remission or succumbed to the disease. Grade I and II acute GVHD was observed in four patients, and, one CML patient survived and all AML patients succumbed in this study (51).

Currently, cord blood transplantation (CBT) is becoming progressively more acceptable and widespread as a therapy for leukemia. CBT utilizes the CIK cells derived from cord blood (CB-CIK) for the immunotherapy of leukemia relapse subsequent to CBT being proven feasible and safe. The CB-CIK cells express high levels of perforin, granzyme and NKG2D. Five patients with AML relapsed following CBT and these patients were treated with a CB-CIK cell infusion, prior to which these patients had undergone additional salvage chemotherapy. In this study, only one patient also experienced grade III GVHD, although this patient experienced a short-term remission. However, all the AML patients succumbed to AML approximately two months subsequent to therapy and it is notable that the GVHD patient demonstrated a response to therapy (52).

In contrast to the aforementioned study, Linn *et al* recently administered allogeneic CIK cells in combination with salvage chemotherapy to 16 patients with hematological malignancy that relapsed subsequent to allo-HSCT and DLI therapy. Overall, there were five patients, including two with ALL, two with HD and one with AML. All five patients achieved complete recession (CR) or major molecular response (MMR) and eventually survived. One AML patient with chromosomal abnormalities in bone marrow cells experienced a second relapse following 10 years of HSCT, while there had been no therapeutic response following DLI infusion and salvage chemotherapy. The patient accomplished the third CR following salvage chemotherapy, with the same therapeutic schedule, and repeated infusion of the allogeneic CIK cells. However, only three patients experienced easily treatable grade I-III GVHD and there was a significantly prolonged time of CR associated with continuous infusion of the CIK cells. The CR time was more than two years and was evidently longer compared with the previous CR time in the follow-up study. This research was the first in the literature to report that allogeneic CIK cells exhibited marked clinical value in leukemia that had undergone previous treatment failure subsequent to DLI therapy and salvage chemotherapy (53). It is inadvisable to adopt the same salvage protocol for all patients as patients may achieve various clinical responses depending on the condition of the patient, including the frequency of relapse, chromosomal abnormalities, gene expression and individual differences.

According to the results of a phase I/II clinical trial in Singapore, 11 patients with AML and 11 patients with CML achieved CR. The AML patients underwent autologous peripheral blood stem cell transplantation and the CML patients were treated with imatinib. Autologous CIK cell immunotherapy exhibited no2benefit in relapse reduction in a four-year follow-up study (54). Nevertheless, results from China reported that a combination of autologous DC-CIK cells and low-dose chemotherapy may benefit patients with non-small cell lung cancer and the CR rate of the CIK cell therapy group was significantly higher compared with the control group (55). Jiang *et al* identified that CIK cells combined with chemotherapy for leukemia significantly improved the CR rate. The 19 patients in the CIK group received a total of 52 courses of CIK cell therapy. The experimental and control group CR rates were 73.4 and 27.3%, respectively. The patients who received more than or equal to three courses of immunotherapy with CIK cells achieved a higher CR rate in a four-year follow-up study (56).

A phase III clinical trial was reported in 2013 by a research group from China that revealed the association between DC-CIK and minimal residual leukemia in a total of 48 acute leukemia patients with hematological CR, but without molecular biological remission. It was found that DC-CIK cells inhibited leukemia gene products, promoted the conversion-negative rate of four-color combination flow cytometric immunophenotype of minimal residual leukemia, facilitated the avoidance of the maximum residue limit and prolonged the remission of the patients. The CR rate of leukemia in the DC-CIK group after three years was 79.2%, while that in the only chemotherapy group was 45.8% (P<0.05) (57).

Benjamin *et al* reported a clinical trial that was performed to evaluate the safety and feasibility of donor-derived CIK cells, using the early infusion of allo-derived CIK cells as consolidative immunotherapy subsequent to non-myeloablative allogeneic transplantation. These patients with MDS were treated by total lymphoid irradiation and rabbit anti-thymocyte globulin, resulting in a two-year actuarial relapse-free survival (RFS) rate and overall survival (OS) rate in the allo-CIK cell group of 62% and 71%, respectively. This was significantly higher compared with the group not treated with CIK infusion, which exhibited a two-year RFS rate of 38% and an OS rate of 41% (57). This demonstrated a significant benefit in the RFS and OS rates associated with allo-derived CIK cell therapy.

From these aforementioned clinical trials and other clinical studies (Table III) (48,52–55,57–59), the common characteristics of clinical CIK cell immunotherapy are easy to identify. Firstly, these studies have demonstrated that autologous, allogeneic or patient-derived CIK cells are a safe, convenient and efficacious therapy in the majority of hematological malignancies. Secondly, the anti-tumor activity of allogeneic CIK cells is stronger compared with autologous CIK cells, infusion of allogeneic CIK cells cause a low incidence of GVHD, which is easily treatable with drugs. Thirdly, allogeneic CIK cells possess important clinical value in the field of hematological malignancy, particularly in maintaining the immune surveillance of leukemia blasts and preventing recurrence. It can be inferred from the current studies that, as for other approaches to immunotherapy, the infusion of CIK cells is likely to be efficacious at disease stages when the tumor burden is relatively low or in an adjuvant setting, rather than in advanced disease. Currently, as uniform, evident standards of evaluating the clinical efficacy of immunotherapy with CIK cells are lacking, it is difficult to accurately assess the long-term clinical benefit for patients associated with allogeneic or autologous CIK cells, alone or in combination with other therapy methods. Support and discussion of the development and standardization of an immunotherapy protocol for leukemia treatment, including expanded times, the tumor response and the combination therapy regimen, is urgently required.

## 4. Future directions

Over previous years, there has been a considerable increase in the recognition of the reticular interactions between leukemia and the human immune system. It is universally acknowledged that immune evasion, including the upregulation of tumor cell-inhibitory molecule expression, plays an important role in tumorigenesis and tumor progression. A current aim is to explore additional effector cells and to use different methods to change the cell membranes of CTLs, alongside chemical receptor and cell vaccine strategies for targeted leukemia therapy. As studies progress, the precise mechanism of the immune response and leukemia stem cell surface markers have been identified, which may aid in further broadening the potential to repackage CIK cells, possibly increasing the anti-tumor activity of these cells. In the near future, CIK cells may be manipulated using the multi-chimeric antigen receptor.

Clinical trials have revealed certain notable results, but uncertain conclusions continue to be reported due to the lack of large-scale, randomized, controlled trials. Schmidt-Wolf *et al* launched initiatives, including an international registry protocol termed the International Registry on CIK Cells to collect the clinical data of patients with cancer who were treated using CIK cells. This may allow for the eventual distribution of a standardized survey and further statistical analysis (55). It can be speculated that CIK cells may establish a larger development space in the field of leukemia immunotherapy, and eventually there may be additional patients who benefit from CIK therapy. However, a series of issues remain to be solved prior to a massive clinical treatment. Firstly, CIK cells require GMP-grade production that takes between six to eight weeks, depending on the quality control release criteria. It is necessary to consider how such a long procedure may correspond with the ideal intervention time to prevent early molecular relapse. Furthermore, in the future, chimeric T-cell receptor engineering of CIK cells may become a more powerful method of leukemia targeting, through the use of CIK cells as a platform. However, it is necessary to note the potential pitfalls of transducing chimeric antigen receptors and methods of improving transducer efficiency according to the present clinical criteria. The use of allo-CIK cells is promising, but only the future may reveal whether this relatively simple form of adoptive cellular immunotherapy may affect the prognosis of patients with hematological malignancies, which remains dismal.

## Figures and Tables

**Table I:** Comparison of various effector cells.

Cells	Cell source	Cell culture	Antitumor activity	MHC restricted	Amplification	GVHD	Reference
LAK	Autologous	IL-2	High	Yes	Hard	NR	9
CD3-AK	Autologous	IL-2, anti-CD3 mAb	Higher	Yes	Easy	NR	28
TIL	Autologous	IL-2	High	Yes	Hard	NR	29
DLI	Allogenic	Leukapheresis	Low	Yes	Hard	High	30
CIK	Autologous or allogenic	IL-2, IFN-γ, anti-CD3 mAb	Highest	No	Easy	Low	31

IL, interleukin; mAb, monoclonal antibody; IFN, interferon; CD, cluster of differentiation; MHC, major histocompatibility complex; GVHD, graft versus host disease; LAK, lymphokine-activated killer; CD3-AK cells, Anti-CD3 antibody-induced activated killer cells; TIL, tumor-infiltrating lymphocytes; DLI, donor lymphocyte infusion; NR, not reported; Hard, 100–1000 times; Easy, 10,000–100,000 times.

**Table II:** Various methods to enhance the lytic activity of CIK cells against leukemia blasts.

Method	Target cells	*In vivo*/*vitro*	Mechanism	Reference
Cytokine				
IL-15 instead of IL-2	MOLT-4, THP-1 and K562	*In vitro*	NKG2D^a^	37
Addition of IL-21		*In vitro*	IL-21R^a^, Perforin^a^, granzyme B^a^, FasL^a^ and activating JAK-STAT	38
Addition of hIFN-α	DSMZ	*In vivo and in vitro*	CB-CIK, CD69^a^ and activation of TRAIL	39
Modified-CIK cells				
IL-24 modified	HL-60	*In vitro*	DC-CIK, CD4^+^/CD25^+^ Tregs^b^	40
Anti-CD19 chimeric receptor	B-ALL	*In vitro*	Anti-CD19 chimeric receptor^a^	41
Anti-CD33 chimeric receptor	Primary AML samples	*In vitro*	Anti-CD33 chimeric receptor^a^, IFN-γ^a^, TNF-α^a^, TNF-β^a^ and IL-2^a^	42
Anti-CD123 chimeric receptor	Primary AML blasts	*In vitro*	Anti-CD123 chimeric receptor^a^	43
Other				
Resveratrol	LSC-like KG-1a	*In vitro*	Target cells NKG2D ligands^a^ and activation of TRAIL	44
Trans-cinnamaldehyde	K562	*In vitro*	Fas/FasL^a^ and mitochondrial transmembrane^b^	45
Anti-CD3 × anti-CD13 BsAb	AML	*In vitro*	BsAb	46
Thymoglobulin	K562	*In vitro*	NK activating/inhibitory receptors, CD158a and CD158b^a^ NKp46^a^, NKG2D^a^ and NKG2A/CD94^a^	47
DC-CIK	Leukemia	*In vitro*	Special targeted minimal residual leukemia cells	48
Tumor vaccines	Tumor	*In vitro*	Enhance the effect of vaccines	49

a Upregulated and

b downregulated.

IL, interleukin; NK, natural killer; JAK-STAT, Janus kinase-signal transducer and activator of transcription; hIFN, human interferon; CB-CIK, cord blood-derived cytokine induced killer cells; CD, cluster of differentiation; TNF, tumor necrosis factor; TRAIL, TNF-related apoptosis-inducing ligand; ALL, acute lymphocytic leukemia; AML, acute myeloid leukemia; BsAb, bispecific antibody; Fas/FasL, CD95 ligand; NKp46, NK cell p46-related protein; NKG2D, natural killer group 2, member D; NKG2A, NKG2, member A; Tregs, regulatory T cells; DC-CIK, dendritic cell-derived CIK cells.

**Table III:** Results from clinical studies using CIK cells as immunotherapy for hematological malignancies.

Cancer	Patients, n	Immunotherapy	Side-effects	Clinical response	Reference
Hematological malignancy	11	Allo-CIK	GVHD (3)	AML CR (1)	52
Hematological malignancy	5	Allo-CIK	LG GVHD (1)	AML PR (1)	53
Hematological malignancy	16	Allo-CIK	LG GVHD (1)	ALL (2), AML (1), HD (2), CR	54
Hematological malignancy	22	CIK	None	No clinical benefit	55
AML and CML	41	CIK vs. CT	None	CCR of CIK vs. control, 73.4 vs. 27.3%^a^	57
Leukemia	48	DC-CIK vs. no CT	None	CCR, DC-CIK vs. no CT: 79.2 vs. 45.8%^a^	48
Myelodysplasia	25	TLI-ATG + allo-CIK vs. TLI-ATG	None	CIK vs. no CIK: Two-year RFS, 62 vs. 38% OS, 71 vs. 41%	58
Multiple myeloma	2	Allo-CIK + bortezomib	None	Significantly improved remission status	59

Numbers in brackets indicate the number of patients affected.

a P<0.05.

CR, complete remission; PR, partial remission; CCR, continuous complete remission; OS, overall survival rate; CT, chemotherapy; RFS, relapse-free survival rate; GVHD, graft versus host disease; LG, low-grade; TTP, time to progression; TLI-ATG, total lymphoid irradiation and rabbit anti-thymocyte globulin; allo, allogenic; CIK, cytokine-induced killer cells; AML, acute myeloid leukemia; ALL, acute lymphocytic leukemia; HD, host disease.
